# Left sided PAPVC with intact IAS—Surgically managed with vertical vein anastomosis to LA appendage: A rare case report

**DOI:** 10.1016/j.ijscr.2019.03.022

**Published:** 2019-03-28

**Authors:** Ganesh Kumar K. Ammannaya, Prashant Mishra, Jayant V. Khandekar

**Affiliations:** Dept. of Cardiovascular & Thoracic Surgery, Lokmanya Tilak Municipal Medical College & General Hospital, Sion, Mumbai, India

**Keywords:** Partial anomalous pulmonary venous connection (PAPVC), Atrial septal defect (ASD), Vertical vein, Left atrial appendage, Case report

## Abstract

Introduction: Partial anomalous pulmonary venous connection (PAPVC) is a rare entity. Only 10% of these are left sided. An intact atrial septum is further uncommon.

Presentation of case: We present a case of left sided PAPVC with no atrial septal defect (ASD), who presented with effort intolerance and an unremarkable physical examination. Computed tomography pulmonary angiography (CTPA) confirmed the primary diagnosis as suggested by an initial 2-D echocardiography, and aided in better understanding of the anatomy.

Conclusion: Patient underwent successful surgery through a simple & reproducible technique of anastomosis of vertical vein to left atrial appendage. Patient recovered uneventfully and was discharged on day 10.

## Highlights

• Rare case of left sided PAPVC with intact interatrial septum.
• Echo diagnosis better characterized with CT imaging.
• Simple and reproducible surgical correction with good result.

## 1 Introduction

PAPVC has a reported incidence between 0.4–0.7% [1]. The most common presentation is a right upper lobe vein draining into either the right atrium or superior vena cava. Only 3% of cases have been reported with drainage from the left lung into the innominate vein. PAPVC most commonly presents with an atrial septal defect (ASD), reportedly in 80–90% of cases. An intact atrial septum is extremely rare [2].

Embryonic development of the pulmonary veins occurs early in cardiovascular development. The prevailing theory is that initial drainage is via the splanchnic plexus into the cardinal and umbilicovitelline veins [1]. A craniocaudal outpouching forms in the sinoatrial region of the heart with extension to the lung buds [1,3]. With caudal regression, the cranial portion develops into the common pulmonary vein, which then incorporates into the left atrial wall [3]. Partial anomalous pulmonary venous return occurs due to failure of connection between the common pulmonary vein and the splanchnic plexus. When occurring in the left upper lobe, a vertical vein is seen collecting the left upper lobe pulmonary veins, and then drains into the brachiocephalic vein [1,3].

Left sided PAPVC forms a left to right shunt, which predisposes the patient to right-sided volume overload, pulmonary hypertension, right ventricular dysfunction and tricuspid regurgitation. In the presence of a concomitant atrial septal defect (ASD), patients may have significant symptoms and present in early childhood. However, isolated left sided PAPVC is often clinically silent until adulthood with incidental diagnosis and vague symptoms [4].

This case report has been reported in line with the SCARE criteria [5].

## 2 Case presentation

**Fig. 1:**
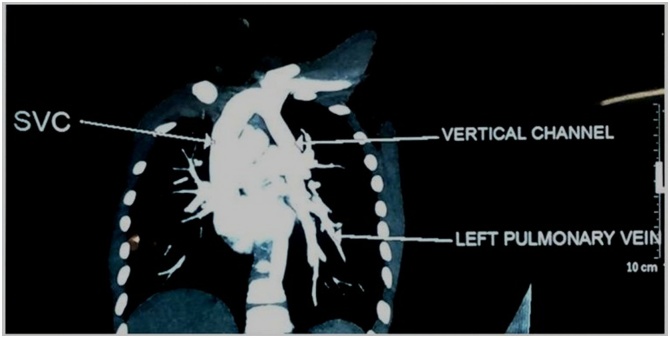
CT Pulmonary Angiography (CTPA) showing the left pulmonary veins joining to form the vertical vein before draining into the superior vena cava (SVC).

**Fig. 2:**
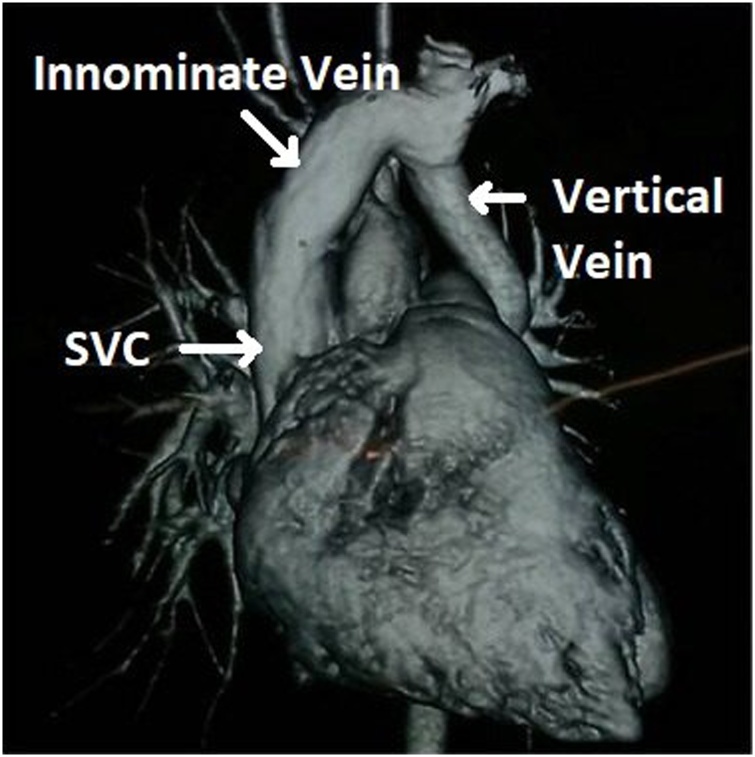
Reconstructed CT Pulmonary Angiography (CTPA) image showing the drainage of the vertical vein into the superior vena cava (SVC) via the innominate vein.

We present the case of a 7 year old boy who presented with effort intolerance and no cyanosis. Clinical examination was unremarkable. Doppler echocardiography revealed left sided pulmonary veins opening into left innominate vein. Right pulmonary veins were seen draining normally into the left atrium. There was no ASD and right atrium and right ventricle were dilated. CTPA (Figs. 1 & 2 ) aided in defining the anatomy. Left pulmonary veins were shown to be joining to form a common channel and draining into superior vena cava via left brachiocephalic vein suggestive of left supracardiac PAPVC thus confirming the preliminary diagnosis of isolated left sided PAPVC.

Pt. was successfully surgically managed. Median sternotomy approach was chosen.

**Fig. 3:**
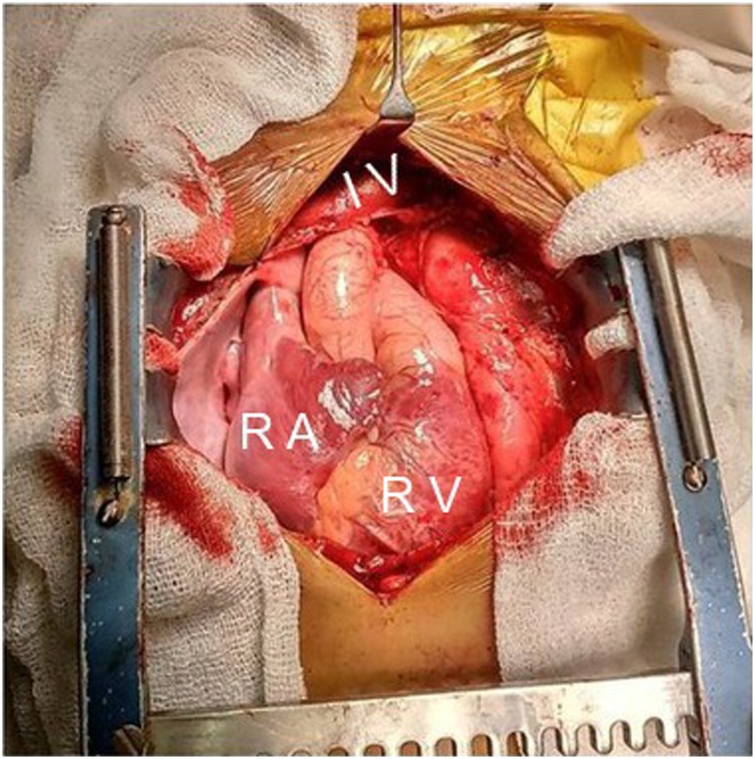
Intraoperative image showing dilated Innominate vein (IV), Right atrium (RA) and Right ventricle (RV).

Innominate vein and superior vena cava were found to be dilated. Also, the right atrium and the right ventricle were dilated (Fig. 3). Vertical vein was seen opening into innominate vein. Left Pulmonary veins were seen opening into the vertical vein. Patient was operated without cardiopulmonary bypass (CPB) support. A 15 mm opening was made in the common chamber horizontally after applying a Cooley’s clamp. Another opening of similar dimension made over LA appendage. Vertical vein was anastomosed to left atrial appendage posteriorly with 6-0 prolene in side-to-side fashion. The vertical vein-innominate confluence was ligated at the end of the procedure. Chest was closed in standard fashion. Mechanical ventilation was required for 12 h. Patient recovered uneventfully and was discharged on Day 10.

## 3 Discussion

Partial anomalous pulmonary venous connection is a rare entity, particularly in the left upper lobe, and even more so with an intact interatrial septum. This condition has potential for significant clinical consequences such as right-sided volume overload, pulmonary hypertension, right ventricular dysfunction and tricuspid regurgitation. Existing imaging modalities contribute to the precise and timely diagnosis of this anomaly. Hioki et al in 1989, reported the successful surgical correction of such a case through thoracotomy, without CPB support, but with the use of polytetrafluoroethylene (PTFE) graft with external ring support and interposing it between the right upper pulmonary vein and the left atrium [6]. Ichihara et al in 1993 reported 2 cases of isolated left PAPVC, one of which was operated through a thoracotomy without CPB support, while the other through median sternotomy with CPB support. Both patients fared well, however, the authors preferred the median sternotomy approach in case of PAPVC not associated with ASD [7]. More recently in 2016, Waqar et al presented a case series of 55 cases of PAPVC, that included a single case of isolated left PAPVC. They approached the case with median sternotomy and CPB support, where the vertical vein that was draining left pulmonary veins to innominate vein was anastomosed side by side with left atrial appendage [8]. Previous surgical case reports [8,9] have shown that, the correction of left sided-PAPVC does carry the risk of complications such as atrial fibrillation, complete heart block, cardiac arrest, and pulmonary venous obstruction. However, no such complication was encountered in our case. We believe the minimal handling of the heart during the performance of the left-sided corrections when done off-pump may be attributed to the minimal morbidity. A table summarizing the various surgical options available for treating left sided PAPVC with and without ASD is summarized in Table 1 [[6], [7], [8], [9], [10]].

**Table 1:** Various techniques in the surgical management of left-sided PAPVC [[6], [7], [8], [9], [10]].

	LEFT-SIDED PAPVC WITH ASD	LEFT-SIDED PAPVC WITH INTACT IAS
With CPB	• Side-to-side left atrio-vertical vein anastomosis on CPB under cardioplegic arrest with closure of ASD through median sternotomy or thoracotomy. • End-to-end or side-to-side left atrial appendage to vertical vein anastomosis on CPB under cardioplegic arrest with closure of ASD through median sternotomy or thoracotomy	• Both side-to-side left atrio-vertical vein anastomosis and end-to-end or side-to-side left atrial appendage to vertical vein anastomosis have been documented through the approaches of either median sternotomy or thoracotomy.
Without CPB	Possible only if – • The ASD is hemodynamically insignificant enough to be left alone; or • If a percutaneous device closure is planned.	• Through thoracotomy with the use of polytetrafluoroethylene (PTFE) graft interposed between the right upper pulmonary vein and the left atrium • Side-to-side or end-to-end anastomosis of left atrial appendage with the vertical vein through median sternotomy or thoracotomy.

We therefore, present our case wherein, we successfully operated a case of isolated left PAPVC through a simple & reproducible technique of a direct anastomosis of vertical vein to left atrial appendage, through median sternotomy, without the need of any prosthetic material or CPB support, with good post-operative outcome.

## 4 Conclusion

This case report describes a rare case of isolated left PAPVC which was managed surgically. Surgical repair without the need of CPB through an easily reproducible technique of median sternotomy and the direct anastomosis of vertical vein to the left atrial appendage represented an effective treatment strategy with good postoperative result.

## Conflicts of interest

No potential conflicts of interest.

## Funding

The case report had no sponsors.

## Ethical approval

This case report is exempt from ethical approval by our institution.

## Consent

Written informed consent was obtained from the parent/guardian of the patient for publication of this case report and accompanying images. A copy of the written consent is available for review by the Editor-in-Chief of this journal on request.

## Author contribution

Ganesh Kumar K Ammannaya - study concept, data collection, surgical therapy for this patient.

Prashant Mishra - surgical therapy for this patient.

Jayant V Khandekar - surgical therapy for this patient.

## Registration of research studies

None.

## Guarantor

Ganesh Kumar K Ammannaya.

## Provenance and peer review

Not commissioned, externally peer-reviewed.
